# PATBox: A Toolbox for Classification and Analysis of P-Type ATPases

**DOI:** 10.1371/journal.pone.0139571

**Published:** 2015-09-30

**Authors:** Dan Søndergaard, Christian Nørgaard Storm Pedersen

**Affiliations:** 1 Bioinformatics Research Centre, Aarhus University, Aarhus, Denmark; 2 Centre for Membrane Pumps in Cells and Disease, Aarhus University, Aarhus, Denmark; International Centre for Genetic Engineering and Biotechnology (ICGEB), INDIA

## Abstract

P-Type ATPases are part of the regulatory system of the cell where they are responsible for transporting ions and lipids through the cell membrane. These pumps are found in all eukaryotes and their malfunction has been found to cause several severe diseases. Knowing which substrate is pumped by a certain P-Type ATPase is therefore vital. The P-Type ATPases can be divided into 11 subtypes based on their specificity, that is, the substrate that they pump. Determining the subtype experimentally is time-consuming. Thus it is of great interest to be able to accurately predict the subtype based on the amino acid sequence only. We present an approach to P-Type ATPase sequence classification based on the *k*-nearest neighbors, similar to a homology search, and show that this method provides performs very well and, to the best of our knowledge, better than any existing method despite its simplicity. The classifier is made available as a web service at http://services.birc.au.dk/patbox/ which also provides access to a database of potential P-Type ATPases and their predicted subtypes.

## Introduction

P-Type ATPases are a large group of transmembrane transporters which pump ions and lipids as part of the regulatory system of the cell. It has been found that the malfunction of some P-Type ATPases cause several severe diseases in humans such as dystonia parkinsonism and Wilson disease [1].

The first P-Type ATPase, the sodium-potassium pump, was discovered in the 1950s and since then over 500 P-Type ATPases have been sequenced, their specificity experimentally verified, and several structures determined [1]. Phylogenetic analysis has shown that the P-Type ATPases can be divided into 5 major and 11 minor subtypes (1A, 1B, 2A, 2B, 2C, 2D, 3A, 3B, 4, 5A and 5B) based on the substrate transported by the pump [2].

Experimentally determining the subtype of a P-Type ATPase is a slow and expensive process, but computational methods for predicting the subtype can aid this analysis significantly. After evaluating several methods for subtype prediction we found that the method presented in this paper provides surprisingly good results and in fact, to the best of our knowledge, performs better than all existing methods despite its simplicity.

## Materials and Methods

We present a machine learning approach for accurately predicting the subtype of a P-Type ATPase from the amino acid sequence by applying the *k*-nearest neighbors (*k*-NN) method [3] to a curated dataset of 515 P-Type ATPase sequences annotated with experimentally verified subtypes. The dataset (S1 Dataset) has been gathered from [2, 4]. The sequences were obtained by mapping the accession identifiers to UniProtKB [5]. Sequences with invalid characters and duplicates were removed. This resulted in 515 sequences with known subtype.

The classifier is made available as a web service. Sequences in FASTA format can be uploaded and the results are available as a web page or can be downloaded in comma-separated values (CSV) format. The web service also provides access to an automatically constructed database of all sequences from UniProtKB containing PROSITE motif D-K-T-G-T-[LIVM]-[TI] (PS00154) characteristic for P-Type ATPases annotated with their classification obtained by our *k*-NN method and a classifier based on the Sequence Learner (SeqL) method [6, 7], which is also made available through the web service. The latter method has previously been applied to P-Type ATPase classification in [8]. This database is thus a valuable resource for exploring P-Type ATPases.

The PATBox web service is implemented in Python using Flask as a web framework, Celery as a job queue, and SQLite as the database. The service is packaged using Docker for reproducability and maintainability.

### Method

The prediction method presented here is based on the *k*-NN method. Given a labeled dataset with data points (*x*_1_, *y*_1_), …, (*x*_*n*_, *y*_*n*_) and a query with unknown label *x*, the *k*-NN method looks at the *k* nearest neighbors to *x* by applying some distance function *d* to each data point in the labeled dataset. The label *y* of *x* is then decided by majority vote. The distance function used in our approach is that of a BLAST [9] search. Thus, for some sequence *x* a search is performed via BLAST and the top *k* results are then used to perform a majority vote. For *k* = 1 this corresponds to a homology search on the curated dataset. Formulating the method in terms of nearest neighbor classification enables us to evaluate it using well-known machine learning evaluation methods.

Additionally we have implemented weighed majority vote such that the weight of a class is given by the sum of the E-values of results belonging to that class divided by the number of results belonging to that class. The class with the minimum weight is chosen as the predicted subtype.

## Results

The overall performance of the *k*-NN classifier has been evaluated by non-stratified 5-fold cross-validation. The dataset is shuffled and split into five parts. A fold is then carried out by training on four parts and predicting on the remaining part. This is repeated five times. We denote this as a run. To obtain an estimate of the variance of the accuracy the run is repeated 20 times, shuffling the sequences every time, for a total of 100 parts per *k* and the standard deviation and average accuracy is reported.

We evaluated both unweighed and weighed *k*-NN for 1 ≤ *k* ≤ 50 to determine the best *k* for each method.

**Fig 1:**
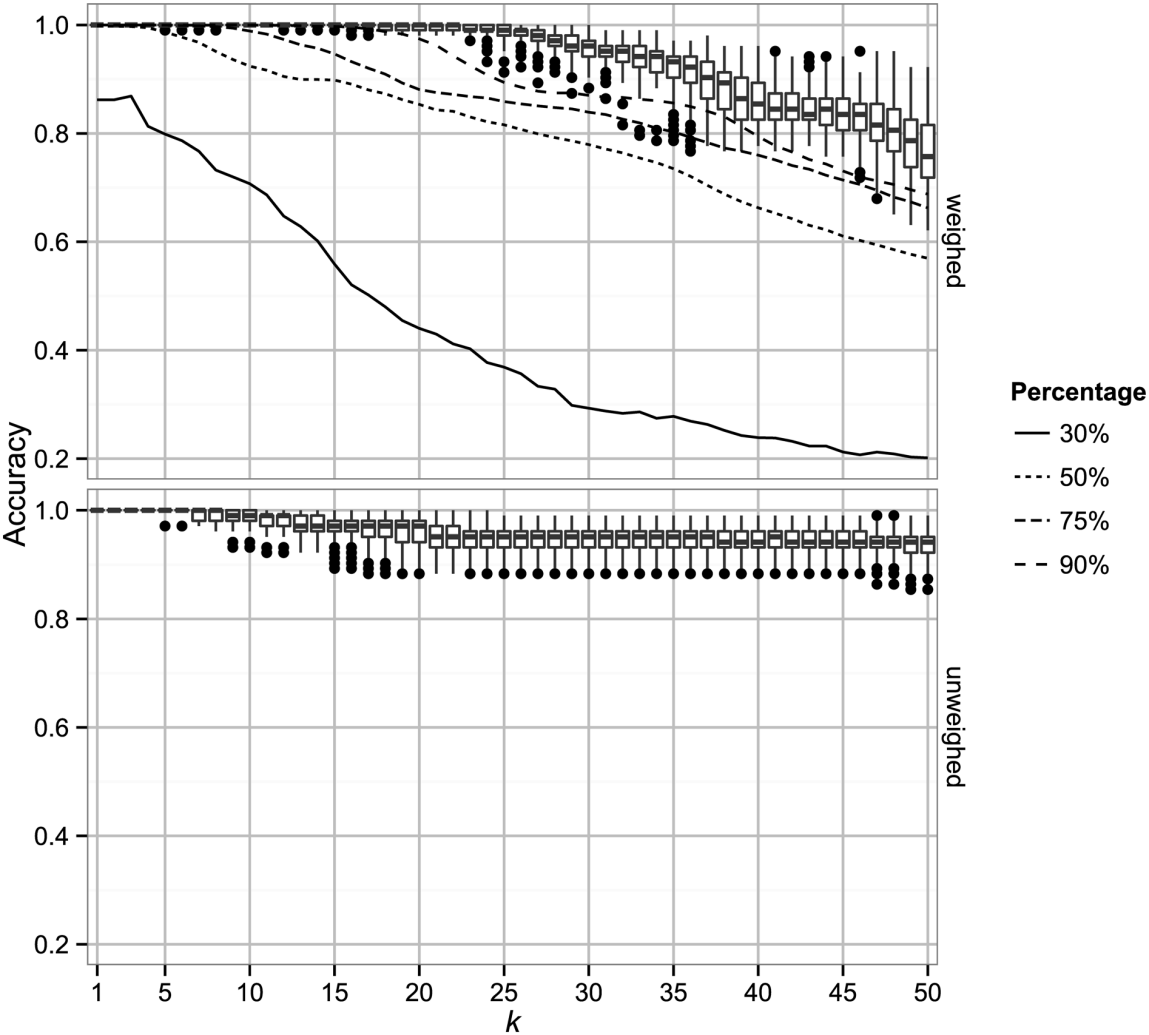
The results of 20 runs of 5-fold cross-validation for 1 ≤ *k* ≤ 50. The weighed and unweighed approaches both perform well for small *k*. For *k* = 1 we obtain an accuracy of 100%. Dots are outliers. Lines show accuracy for reduced datasets.

The results are summarized in Fig 1 as a box plot. The average accuracy of the shuffled and repeated folds for each majority vote method is shown on the vertical axis with error bars showing the standard deviation. As we expect the accuracy of the two methods for *k* = 1 is the same. For both weighed and unweighed majority vote we see that as *k* increases as the accuracy decreases and the standard deviation increases. We obtain the best result when *k* = 1 for which the accuracy is 100%. Similar results are obtained for 2-fold cross-validation, where only half of the data is available for training, suggesting that the classifier is not prone to over-fitting (data not shown).

The high accuracy is not a surprise. The average area-under-curve (AUC) over all classes of the Structured Logistic Regression (SLR) classifier in [8] is 97.7%. An advanced prediction method presented in [10] based on neural networks also yields a very high accuracy of 99.1% based on a 10-fold cross-validation on 5/6 of the dataset.

The consistently good results obtained through a variety of independent methods also suggests that the methods are not over-fitting and should generalize well.

To further investigate the predictive power of the *k*-NN method we used the CD-HIT [11] web server to cluster the dataset at similarity thresholds of 30%, 50%, 75% and 90%, and extracted the representative sequences of each cluster. The cross-validation was repeated with weighed *k*-NN on the four reduced datasets and the results are shown as lines in Fig 1 (error bars omitted to reduce complexity of the plot). We find that the method is very robust, obtaining 100% accuracy for k = 1 for similarity thresholds as low as 50%.

## Discussion

We present a method for accurate classification of P-Type ATPases into 11 subtypes based on the *k*-NN method using BLAST as a distance measure. We show experimentally that the optimal *k* = 1 for which we obtain an accuracy of 100%. More advanced methods have previously provided similar results which leads us to believe that the representative sequences for each subtype in the dataset cluster well based on sequence similarity. The results obtained by the *k*-NN method confirms this observation.

The contribution of this paper is twofold. Firstly, we show that *k*-NN performs extremely well on P-Type ATPases, despite the simplicity of the method, and that homology searches therefore can be used to determine the subtype of P-Type ATPase sequence. Secondly, the method presented here performs better than a multitude of more complicated methods, emphasising that simple methods should not be forgotten, even in the presence of more complicated methods.

The classifier is made available through a new web service for researchers in the field of P-Type ATPases, the P-Type ATPase Toolbox (PATBox), which also gives access to a database of predicted P-Type ATPases and their predicted subtype, based on UniProtKB [5].

## Supporting Information

S1 Dataset Dataset of annotated P-Type ATPase sequences. The dataset used for cross-validation and final training of the classifier described in this manuscript.
(FASTA)

## References

[1] Bublitz M, Morth JP, Nissen P. P-type ATPases at a glance. Journal of Cell Science. 2011;124(15):2515–2519. 21768325 10.1242/jcs.088716

[2] Axelsen KB, Palmgren MG. Evolution of Substrate Specificities in the P-Type ATPase Superfamily. Journal of molecular evolution. 1998;46(1):84–101. 10.1007/PL00006286 9419228

[3] Cover T, Hart P. Nearest neighbor pattern classification. IEEE Transactions on Information Theory. 1967;13 10.1109/TIT.1967.1053964

[4] Møller AB, Asp T, Holm PB, Palmgren MG. Phylogenetic analysis of P5 P-type ATPases, a eukaryotic lineage of secretory pathway pumps. Molecular Phylogenetics and Evolution. 2008;46(2):619–634. 10.1016/j.ympev.2007.10.023 18155930

[5] Magrane M, Consortium UP. UniProt Knowledgebase: A hub of integrated protein data. Database. 2011;2011 10.1093/database/bar009 21447597 PMC3070428

[6] Ifrim G, Bakir G, Weikum G. Fast logistic regression for text categorization with variable-length n-grams. In: the 14th ACM SIGKDD international conference. New York, New York, USA: ACM, Association for Computing Machinery; 2008. p. 354–362.

[7] Ifrim G, Wiuf C. Bounded coordinate-descent for biological sequence classification in high dimensional predictor space. In: KDD’11: Proceedings of the 17th ACM SIGKDD international conference on Knowledge discovery and data mining. ACM Request Permissions; 2011. p. 708–716.

[8] Pedersen BP, Ifrim G, Liboriussen P, Axelsen KB, Palmgren MG, Nissen P, et al Large Scale Identification and Categorization of Protein Sequences Using Structured Logistic Regression. PLoS ONE. 2014;9(1):e85139 10.1371/journal.pone.0085139 24465495 PMC3896382

[9] Camacho C, Coulouris G, Avagyan V, Ma N, Papadopoulos J, Bealer K, et al BLAST plus: architecture and applications. BMC Bioinformatics. 2009;10:–. 10.1186/1471-2105-10-421 20003500 PMC2803857

[10] Espeholt L. Neural Networks for Classification of Protein Sequences. M.Sc. Thesis, Aarhus University. 2014.

[11] Huang Y, Niu B, Gao Y, Fu L, Li W. CD-HIT Suite: A web server for clustering and comparing biological sequences. Bioinformatics. 2010;26(5):680–682. 10.1093/bioinformatics/btq003 20053844 PMC2828112

